# Aortic Arch Phenotypes in Double Outlet Right Ventricle (DORV)—Implications for Surgery and Multi-Modal Imaging

**DOI:** 10.3390/jcdd9080262

**Published:** 2022-08-12

**Authors:** Alessandro Gressani, Renata Aynetdinova, Martin Kostolny, Silvia Schievano, Andrew Cook, Georgios Belitsis

**Affiliations:** 1 Medical School, University College London, London WC1E 6DE, UK; 2 Department of Children’s Cardiovascular Disease, University College London, London WC1N 1DZ, UK; 3 Cardiothoracic Surgery Department, Great Ormond Street Hospital for Children, London WC1N 1DZ, UK

**Keywords:** aortic arch, patch aortoplasty, tubular hypoplasia, double outlet right ventricle, DORV, virtual reality, 3D printing

## Abstract

Abnormal aortic arches (AAAs) cover a spectrum of malformations, including abnormal laterality, branching patterns, and flow-limiting narrowing, which themselves vary from tubular hypoplasia, through discrete coarctation, to complete interruption of the arch. Neonatal surgery within the first days of life is necessary for most of these morphologies. Patch aortoplasty is widely used as it can offer a good haemodynamic result, being tailored to each combination of presenting pathologies. Our study hypothesis was that arch malformations are frequent in DORV and exhibit a plethora of phenotypes. We reviewed 54 post-mortem heart specimens from the UCL Cardiac Archive, analysing morphological features that would potentially influence the surgical repair, and taking relevant measurements of surgical importance. AAAs were found in half of the specimens, including 22.2% with aortic arch narrowing. In total, 70% and 30% of narrow arches had a subpulmonary and subaortic interventricular defect, respectively. Z-scores were significantly negative for all cases with tubular hypoplasia. We concluded that arch malformations are a common finding among hearts with DORV. Surgery on the neonatal aortic arch in DORV, performed in conjunction with other interventions that aim to balance pulmonary to systemic flow (Qp/Qs), should be anticipated and form an important part of multi-modal imaging.

## 1. Introduction

Commonly occurring structural malformations of the aortic arch include a right aortic arch, a double arch, retro-oesophageal subclavian arteries, and a left common carotid artery arising from the brachiocephalic artery [1].

Flow-limiting lesions exist on a spectrum of severity, from mild narrowing and coarctation to atresia or complete interruption. Aortic coarctation accounts for 6–8% of all congenital heart diseases [2].

### 1.1. Management

All reparative approaches in the neonate are surgical. Neonatal patch aortoplasty with pulmonary homograft is often the approach of choice, guaranteeing low re-operation and mortality rates [3]. The homograft is often procured from the floor of the left pulmonary artery due to its innate curvature.

Along with aortoplasty, patch design is a key indicator of surgical success, where incorrect sizing is a major factor in re-coarctation [4]. Patch sizing follows empirical methods, aiming to be both concave and convex, mimicking the saddle shape of the arch [4].

The surgical focus on hearts with DORV has always been associated with the interventricular defect (IVD) [5]. Historically, complex DORV could have been a candidate for univentricular palliation. Biventricular repair of complex DORV [6] relies on the tunnelling of blood from the left ventricle to the outflow tract of choice [7,8]. Local coordinates of the outflow tracts to the IVD are the determining factor in choosing between solitary intracardiac repair and a baffle to the MPA root and ASO [9,10]. If coupled with arch augmentation, repair is a major surgical challenge, with longer bypass, cardiac, and circulatory arrest times. The up-to-now unmapped phenotypic variants of arch malformations observed in DORV may increase the risk of such a procedure, potentially influencing the outcome.

### 1.2. Aims and Hypothesis

Advancement of patch aortoplasty for effective and low-risk surgery relies on optimal planning. We studied aortic arch morphology in post-mortem heart specimens with DORV. We hypothesised that arch anomalies requiring surgery within the first days of life are a common finding among DORV and are diverse in nature. Our aim was to prove that phenotypes of arch pathology are highly variable, meriting customised surgical augmentation techniques.

## 2. Materials and Methods

### 2.1. Study Design

We reviewed 54 post-mortem heart specimens from the UCL Cardiac Archive, held at the Zayed Centre for Research, London, UK. We selected specimens coded as DORV in the archive’s database, confirming diagnosis on examination.

### 2.2. Gross Examination

We examined all hearts using the process of sequential segmental analysis [11]. We reviewed the arch orientation and branching pattern and assessed for evidence of narrowing. Arch narrowing was characterised as one from: isolated isthmic aortic coarctation; aortic arch hypoplasia; atresia; or aortic arch interruption.

Cases were examined by two observers: a medical student and a surgeon, with the occasional consultation of a highly experienced cardiac morphologist.

Intact vessels remained unopened, and their diameter was measured by having them flattened against a ruler. For the arches that have been previously incised and were open, we used a silk-tie and ruler technique to measure the inner circumference. The diameter was obtained using the formula: diameter = circumference/π.

Additional measurements in several narrow-arched specimens were taken as an example of invaluable data points for patch sizing.

**Figure 1:**
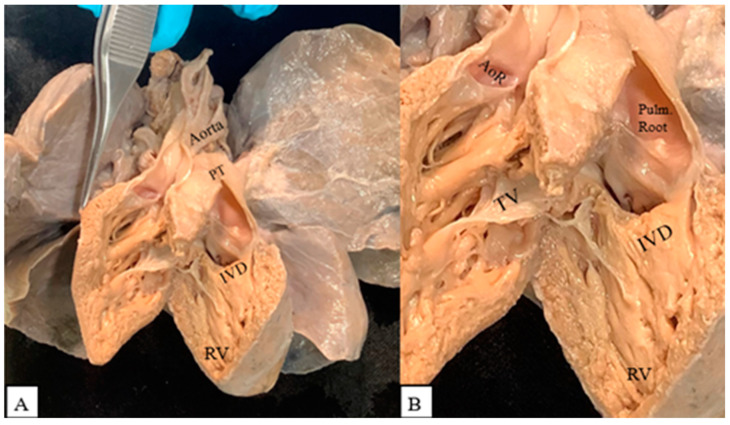
(**A**) Double outlet right ventricle with subpulmonary interventricular defect (IVD). PT: pulmonary trunk; RV: right ventricle. (**B**) Both arterial roots arising from the right ventricle. AoR: aortic root; Pulm. Root: pulmonary root; TV: tricuspid valve.

We coded the IVD in relation to its proximity to the arterial roots [5]. A case that clearly demonstrates a subpulmonary IVD is shown in Figure 1.

The aortic arch was defined as hypoplastic if its transverse diameter was less than the control arch diameter. The control arch diameter was estimated as mean bodyweight at birth +1 mm, using the formula described by Karl et al. [12]. The ‘mean bodyweight’ for the above calculation was the calculated mean bodyweight of male and female children, as derived from growth charts of newborns (40 weeks’ gestation) at the 50th percentile [13]. The average control weight and height were 3.5 kg and 50.5 cm, respectively.

### 2.3. Data Analysis

For specimens found to have arch narrowing, we used an online clinical tool [14] to estimate z-scores of distal arch diameter and isthmus diameter. Along with our measurements of arch diameter, we used height in cm and weight in kg of term neonates in the 50th percentile, taking the mean of males and females [13]. We selected the female gender on the calculator tool. A z-score of −2 or less was deemed to constitute hypoplasia [15], with a likely significant effect on haemodynamics, warranting surgery within the first days of life.

We calculated confidence intervals (CIs) of proportions using an online calculator tool [16] at a 95 % confidence level.

## 3. Results

### 3.1. Study Profile

**Figure 2:**
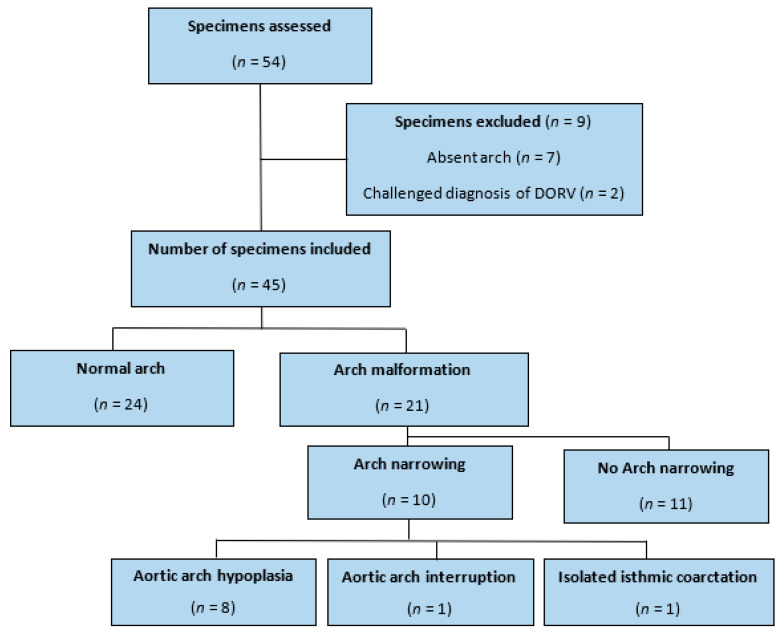
Study profile. Specimens with arch malformations showed one or multiple from: a right arch; mirror image branching pattern of epi-aortic branches; presence of aberrant branch(es); common or trunk origin of branch(es); arch narrowing. Narrow arches showed one or multiple from: aortic arch hypoplasia; isolated isthmic coarctation; aortic arch interruption.

Figure 2 summarises our study profile. Of the 54 selected specimens with DORV, 9 were excluded. In seven cases, the aortic arch had been removed in whole or in part, while in two cases, we challenged the documented database diagnosis of DORV.

Among the 45 remaining specimens, 21 (46.6%) had an arch malformation (95% CI of proportions: 32–61.2%) and 10 (22.2%) presented with arch narrowing (95% CI prop: 10.1–34.3%). Eight (17.7%) presented with hypoplasia of the distal arch (95% CI prop: 6.5–28.9%), one (2.2%) with isolated aortic coarctation (95% CI prop: 0–6.5%), and one (2.2%) with IAA (95% CI prop: 0–6.5%). The one case with isolated isthmic coarctation also had an aberrant right subclavian branch.

### 3.2. Laterality and Branching of the Aortic Arch

**Figure 3:**
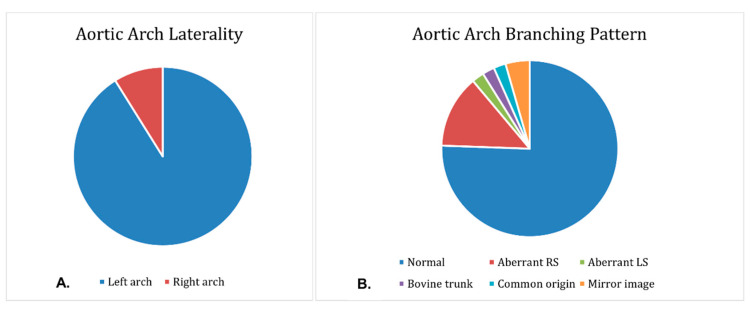
Pie charts showing (**A**) aortic arch laterality and (**B**) aortic arch branching patterns.

Specimens exhibited a variety of phenotypes. The aortic arch was on the left in 41 (91.1%) specimens (95% CI prop: 82.8–99.4%) and on the right in 4 (8.9%) cases (95% CI prop: 0.6–17.2%). In total, 34 (75.6%) hearts had a normal branching pattern (95% CI prop: 63.1–88.1%), while 6 (13.3%) had an aberrant right subclavian branch (95% CI prop: 3.4–23.2%). One (2.2%) had an aberrant left subclavian artery (95% CI prop: 0–6.5%) and was also a right arch. One (2.2%) specimen had a bovine trunk (95% CI prop: 0–6.5%), and one (2.2%) had a common origin of all epi-aortic branches (95% CI prop: 0−6.5%). Two (4.4%) hearts exhibited mirror image branching (95% CI prop: 0–10.4%) and were both right arches. Figure 3 presents the variation in the laterality and branching patterns of the arch.

### 3.3. Aortic Arch Narrowing

**Figure 4:**
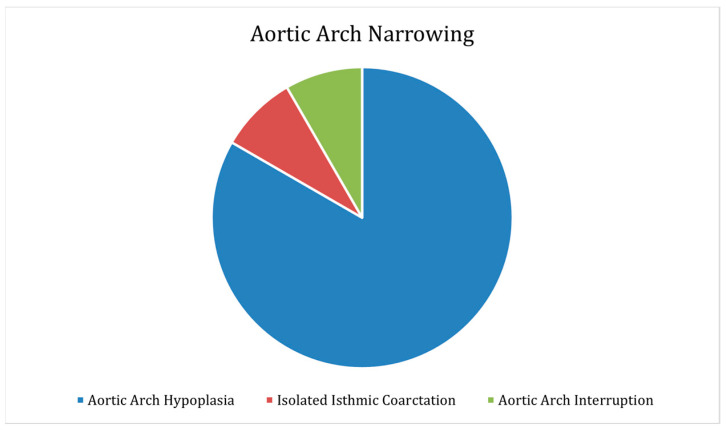
Aortic arch narrowing, including aortic arch hypoplasia, isolated isthmic coarctation, and aortic arch interruption.

Ten (22.2%) specimens had aortic arch narrowing (95% CI prop: 9.9–34.1%), as shown in Figure 4. Eight (17.7%) exhibited aortic arch hypoplasia (95% CI prop: 6.5–28.9%), one (2.2%) showed isolated isthmic coarctation (95% CI prop: 0–6.5%), and one (2.2%) showed aortic arch interruption (95% CI prop: 0–6.5%). All 10 (100%) narrow arches were left-oriented. Out of the narrow arches, nine (90%) had a normal branching pattern (95% CI prop: 71.4–100%), and one (0.1%) had an aberrant right subclavian branch (95% CI prop: 0–28.6%).

### 3.4. Interventricular Defects (IVD)

**Figure 5:**
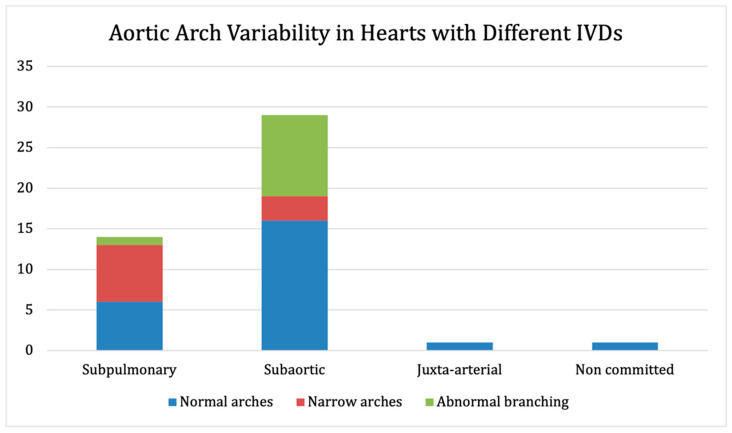
Arch variability in hearts with different interventricular communications.

In total, 14 (31.1%) hearts had a subpulmonary IVD (95% CI prop: 17.6–44.6%), 29 (64.4%) had a subaortic IVD (95% CI prop: 50.4–78.4%), 1 (2.2%) had a juxta-arterial IVD (95% CI prop: 0–6.5%), and 1 (2.2%) had a non-committed IVD (95% CI prop: 0–6.5%). Out of the 10 hearts with aortic arch narrowing, 7 (70%) had a subpulmonary IVD (95% CI prop: 41.6–98.4%), and 3 (30%) had a subaortic IVD (95% CI prop: 1.6–58.4%). Out of the 11 arches with branching abnormalities, 1 (9.1%) had a subpulmonary IVD (95% CI prop: 0–26.1%), and 10 (90.9%) had a subaortic IVD (95% CI prop: 73.9–100%). Figure 5 shows the aortic arch variability among hearts with different interventricular communications.

### 3.5. Data Analysis

For nine specimens with aortic arch narrowing, the distal arch diameter and isthmus diameter were used to calculate z-scores of the distal arch and isthmus, respectively. The case with aortic arch interruption was excluded. All eight specimens with aortic arch hypoplasia had z-scores lower than −2 for both the distal arch and isthmus (the latter was missing in three cases and therefore no z-score was calculated). The case with isolated isthmic coarctation had a positive z-score for the distal arch (3.15) and a negative z-score for the isthmus (−1.03). Table 1 shows the above findings. 

## 4. Discussion

DORV is an uncomplicated cardiac malformation, if defined simply with both arterial trunks arising predominantly from the morphologically right ventricle, but it is remarkably diverse in terms of detailed morphology and likely developmental mechanisms. Malformation of the interventricular septum and the outflow tract of the right ventricle is an often-reported component of genetically diverse animal models [17,18]. Aiming towards the identification of broader developmental pathways involving the distal aortic arches might help in explaining the results of our study, reflecting the diagnostic and surgical challenges reported in humans.

A patient-specific approach to DORV is the norm in major paediatric cardiology centres across the world, often involving the creation of 3D models during pre-surgical planning (3D printing, VR, etc.). While the focus is typically on the approach to intraventricular repair, our impression, and hypothesis, was that aortic arch anomalies are commonly seen in DORV and should not be discounted during multi-modal imaging and surgical repair.

Our systematic approach to specimen examination and data collection resulted in reliable findings in support of our hypothesis, linking half of the DORV cases to aortic arch malformations. In our study, we determined that the aortic arch had developed abnormally in nearly one half of the DORV specimens, and in nearly one quarter, a surgically relevant narrowing was evident. This is of translational value, not only in terms of imaging and surgical repair, but also in terms of deciphering developmental mechanisms.

A distal arch diameter of 4 mm or less was documented in 8 out of 45 cases in our study. Repair of such a lesion would require patch aortoplasty involving the use of a heart–lung machine and profound hypothermia. This is a profoundly different and major procedure when compared to a conventional repair of isolated aortic coarctation performed through thoracotomy.

Traditionally, aortic arch narrowing is recognised as a feature of DORV and a subpulmonary IVD, due to malalignment of the outlet (conal) septum towards the subaortic outflow. The similar distribution of arch malformations in hearts with subpulmonary and subaortic IVDs in our study suggests the picture is more complex than this and supports the need for truly customised approaches to surgical repair, as well as focused imaging for all DORV cases.

In DORV repair, the systemic ventricle is baffled via the IVD to the systemic semilunar (aortic) valve. This can be challenging in patients with a remote VSD, prominent subaortic conus, or abnormal attachments of AV valves. Subpulmonary VSDs can be baffled towards the pulmonary valve with concomitant arterial switch operation [8,10].

Given the increased likelihood of arch narrowing in DORV in this study, careful prenatal and early postnatal examination of the aortic arch in all DORV patients is warranted in order to exclude arch pathology prior to postnatal closure of the arterial duct.

### 4.1. Limitations

The relatively small sample size justifies the need to repeat this morphological study across multiple archives or cardiac registries. Application of a single measuring technique (sizing intact arch against a ruler) was impossible due to individual anatomy and previous dissection. The use of post-mortem tissue is accompanied by the caveat that organs are always subject to change after fixation. This was a post-mortem study and therefore our cases may be considered to represent the more severe end of the spectrum of arch malformation. Nonetheless, more than half of our arch anomalies were non-obstructive and, while important to consider surgically, are unlikely to have resulted in severe haemodynamic compromise, leading to death.

### 4.2. Future Directions

Our study proved the association of major vascular malformations and an already surgically challenging congenital heart disease. The careful investigation of DORV arches prenatally and postnatally will help in early identification of the pathology and allow for planning of reparative surgery. In recent years, there have been strong arguments in favour of 3D printing and, more recently, VR [19,20,21] due to their potential usefulness in increasing understanding and planning of complex DORV surgery.

There are pilot studies suggesting that patch tailoring can be supported by computational modelling [22] and 3D printing. A 3D print of a VR-created patch can be used as a pattern for cutting out patches in the operating room.

Further, 3D printing of the ultrastructure of vascular intracellular stroma with the use of biocompatible-bioabsorbable ‘ink’ can be used in the creation of patches that will act as scaffolds for human cell growth.

Enhancement of diagnostic tools for arch pathology may be achieved via artificial intelligence. The arch malformations and discrete pathologies recognised in our study, if enhanced, could aid in the development of diagnostic algorithms. These algorithms should be able to identify all arch anomalies including an atypical orientation, branching, and luminal size in different segments. Data sets of a more detailed and arch-oriented morphological mapping of DORV cases will be an invaluable step in the creation of machine learning tools that will support the above.

## 5. Conclusions

Our study found a significant occurrence of aortic arch malformations in hearts with DORV. An atypical orientation, branching pattern, and size of the arch are common and should be expected. The combination of intracardiac repair and arch augmentation represents a surgical challenge and should be approached in an individualised fashion. Augmented reality [19,20,21] and 3D printing [20,23] can be used for planning and patch design in patch aortoplasty procedures [24]. Finally, artificial intelligence [25,26] tailored to recognise arch pathology could support paediatric cardiologists in pre- and postnatal diagnosis.

## Figures and Tables

**Table 1:** Diameter and z-scores of the distal arch and isthmus for 9 specimens with aortic arch narrowing. The 1 case of aortic arch interruption was excluded. In 2 hearts, the isthmus had been removed, and in 1, it had been repaired, so no z-score was calculated.

Diagnosis	Distal Arch Diameter	Z-Score of Distal Arch	Isthmus Diameter	Z-Score of Isthmus
Isolated isthmic coarctation	9	3.24	4	−1.03
Aortic arch hypoplasia	4	−3.30	Isthmus removed	-
Aortic arch hypoplasia	3	−5.62	EEA coarctation repaired at isthmus	-
Aortic arch hypoplasia	4	−3.30	3	−3.00
Aortic arch hypoplasia	4	−3.30	3	−3.00
Aortic arch hypoplasia	4	−3.30	Isthmus removed	-
Aortic arch hypoplasia	2	−8.89	3	−3.00
Aortic arch hypoplasia	4	−3.30	3	−3.00
Aortic arch hypoplasia	2.8	−6.18	2	−5.78

## Data Availability

Not applicable.

## References

[1] Arnaiz-Garcia M.E., González-Santos J.M., López-Rodríguez J., Dalmau-Sorlí M.J., Bueno-Codoñer M., Arévalo-Abascal A., García-Hierro F., Arnáiz-García A.M., Arnáiz J. (2014). A bovine aortic arch in humans. Indian Heart J..

[2] Ringel R.E., Gauvreau K., Moses H., Jenkins K.J. (2012). Coarctation of the Aorta Stent Trial (COAST): Study design and rationale. Am. Heart J..

[3] Tremblay D., Zigras T., Cartier R., Leduc L., Butany J., Mongrain R., Leask R.L. (2009). A comparison of Mechanical Properties of Materials Used in Aortic Arch Reconstruction. Ann. Thorac. Surg..

[4] Hasegawa T., Oshima Y., Maruo A., Matsuhisa H., Tanaka A., Noda R., Matsushima S. (2015). Aortic arch geometry after the Norwood procedure: The value of arch. J. Thorac. Cardiovasc. Surg..

[5] Ebadi A., Spicer D.E., Backer C.L., Fricker F.J., Anderson R.H. (2017). Double-outlet right ventricle revisited. J. Thorac. Cardiovasc. Surg..

[6] Stark J.F., De Leval M.R., Tsang V.T. (2006). Surgery for Congenital Heart Defects.

[7] Mostefa-Kara M., Houyel L., Bonnet D. (2018). Anatomy of the ventricular septal defect in congenital heart defects: A random association?. Orphanet. J. Rare Dis..

[8] Anderson R.H., McCarthy K., Cook A.C. (2001). Double outlet right ventricle. Cardiol. Young.

[9] Lacour-Gayet F. (2008). Intracardiac repair of double outlet right ventricle. Semin. Thorac. Cardiovasc. Surg. Pediatric Card. Surg. Annu..

[10] Lacour-Gayet F., Haun C., Ntalakoura K., Belli E., Houyel L., Marcsek P., Wagner F., Weil J. (2022). Biventricular repair of double outlet right ventricle with non-committed ventricular septal defect (VSD) by VSD rerouting to the pulmonary artery and arterial switch. Eur. J. Cardio-Thorac. Surg..

[11] Anderson R.H., Ho S.Y. (1997). Continuing Medical Education. Sequential segmental analysis—Description and categorization for the millennium. Cardiol. Young.

[12] Karl T.R., Sano S., Brawn W., Mee R.B. (1992). Repair of hypoplastic or interrupted aortic arch via sternotomy. J. Thorac. Cardiovasc. Surg..

[13] RCPCH (2021). Neonatal and Infant Close Monitoring Growth Chart. https://www.rcpch.ac.uk/sites/default/files/Girls_neonatal_and_infant_close_monitoring_growth_chart.pdf.

[14] Dyar D. (2020). Z-Scores and Reference Values for Pediatric Echocardiography: Aortic Arch. http://www.parameterz.com/sites/aortic-arch.

[15] Jonas R.A. (2013). Comprehensive Surgical Management of Congenital Heart Disease.

[16] Confidence Interval for Proportion Calculator—MathCracker.com. https://mathcracker.com/confidence-interval-proportion-calculator.

[17] Stefanovic S., Etchevers H.C., Zaffran S. (2021). Outflow Tract Formation-Embryonic Origins of Conotruncal Congenital Heart Disease. J. Cardiovasc. Dev. Dis..

[18] Verzi M.P., McCulley D.J., De Val S., Dodou E., Black B.L. (2005). The right ventricle, outflow tract, and ventricular septum comprise a restricted expression domain within the secondary/anterior heart field. Dev. Biol..

[19] Milano E.G., Pajaziti E., Sauvage E., Cook A., Schievano S., Mortensen K., Taylor A.M., Marek J., Kostolny M., Capelli C. (2019). Taking Surgery Out of Reality. Circ. Cardiovasc. Imaging.

[20] Belitsis G. Case 1. Advanced 3D modelling and augmented reality in congenital heart surgery—Case 1-DORV. Proceedings of the EACTS Conference.

[21] Kostolny M. (2021). Preparation for Surgery Using VR’. Multimodal Imaging in Complex Double Outlet Right Ventricle. https://ucl.zoom.us/j/95769543223?pwd=Z2x3TVZuZzdhNXEzcHIvWCtvMkZWZz09.

[22] Aynetdinova R. (2021). The Potential Use of Augmented Reality and 3D Printing in the Planning of Patch Arterioplasty Procedures, for Neonatal and Infantile Congenital Cardiac Disease.

[23] Milano E.G., Capelli C., Wray J., Biffi B., Layton S., Lee M., Caputo M., Taylor A.M., Schievano S., Biglino G. (2019). Current and future applications of 3D printing in congenital cardiology and cardiac surgery. Br. J. Radiol..

[24] Dent C. (2022). Use of 3D Models in Communicating the Native and Operative Anatomy of Highly Complex and Rare Congenital Heart Disease.

[25] Arnaout R., Curran L., Zhao Y., Levine J.C., Chinn E., Moon-Grady A.J. (2021). An ensemble of neural networks provides expert-level prenatal detection of complex congenital heart disease. Nat Med..

[26] Yeo L., Romero R. (2013). Fetal intelligent navigation echocardiography (FINE): A novel method for rapid, simple, and automatic examination of the fetal heart. Ultrasound Obstet. Gynecol..

